# Engineered Microorganisms as GLP-1-Oriented Living Therapeutics for Diabetes and Obesity: Mechanisms and Translational Challenges

**DOI:** 10.3390/ijms27219977

**Published:** 2026-11-07

**Authors:** Arthur A. Lee, Victoria A. Khotina, Olesya O. Klychkova, Vitalia S. Novikova, Dmitry A. Kashirskikh, Vagif A. Gasanov

**Affiliations:** Koltzov Institute of Developmental Biology of the Russian Academy of Sciences, 119334 Moscow, Russia; a.a.lee@idbras.ru (A.A.L.); o.o.klychkova@idbras.ru (O.O.K.); v.s.novikova@idbras.ru (V.S.N.); d.a.kashirskikh@idbras.ru (D.A.K.); v.a.gasanov@idbras.ru (V.A.G.)

**Keywords:** engineered microorganisms, living therapeutics, glucagon-like peptide-1, GLP-1 receptor agonists, type 2 diabetes mellitus, obesity, enteroendocrine signaling, intestinal delivery

## Abstract

Glucagon-like peptide-1 receptor agonists (GLP-1RAs) are established treatments for obesity and type 2 diabetes mellitus, but gastrointestinal barriers constrain oral delivery of peptide therapeutics. Genetically engineered microorganisms have been investigated as intestinal sources of GLP-1-related therapeutic molecules. These platforms produce GLP-1 or GLP-1-derived peptides, express modified GLP-1RAs or fusion proteins, or release effectors that stimulate endogenous GLP-1 secretion. Preclinical studies have reported improvements in glycemic measures and, in some models, reductions in body weight or weight gain. However, recombinant product expression and treatment-associated effects are more consistently demonstrated than exposure to intact biologically active products in vivo, receptor or pathway dependence, and the contribution of the engineered construct relative to the microbial chassis. Proteolysis, mucus interactions, restricted epithelial permeability, and receptor localization constrain access to intestinal and systemic targets. This review critically evaluates the preclinical evidence for GLP-1-oriented engineered microorganisms, with emphasis on intestinal target accessibility, local and systemic mechanisms, microbial chassis and genetic design, and the safety, manufacturing, and regulatory requirements for clinical translation.

## 1. Introduction

Obesity and type 2 diabetes mellitus (T2DM) represent major global health burdens associated with increasing prevalence and shared metabolic disturbances. More than one billion people were living with obesity in 2022 [1], and an estimated 589 million adults aged 20–79 years were living with diabetes in 2024, predominantly T2DM [2,3,4,5]. Obesity and T2DM are closely linked through adipose tissue dysfunction, insulin resistance, ectopic lipid deposition, chronic low-grade inflammation, and progressive pancreatic β-cell dysfunction [6,7,8,9].

Glucagon-like peptide-1 receptor agonists (GLP-1RAs) are established treatments for T2DM and obesity. Activation of the glucagon-like peptide-1 receptor (GLP-1R) enhances glucose-dependent insulin secretion, suppresses glucagon secretion during hyperglycemia, delays gastric emptying, and reduces appetite, contributing to improved glycemic control and weight reduction [10,11,12,13,14]. Native glucagon-like peptide-1 (GLP-1) is rapidly cleaved by dipeptidyl peptidase-4 (DPP-4) and cleared from the circulation, resulting in a plasma half-life of only a few minutes [14]. Long-acting GLP-1RAs extend the duration of biological activity through amino acid substitutions, acylation, albumin binding, or other structural modifications, but most require subcutaneous administration [15,16]. Oral semaglutide demonstrates the feasibility of systemic oral delivery of a peptide agonist but requires co-formulation with the absorption enhancer salcaprozate sodium (SNAC). Its absolute bioavailability is low (approximately 1%) and variable, with absorption occurring primarily across the gastric epithelium [17,18]. These characteristics illustrate the physiological barriers associated with systemic oral delivery of peptide therapeutics.

Genetically engineered microorganisms provide an alternative approach in which viable microbial cells are designed to produce therapeutic molecules within the gastrointestinal tract [19,20,21]. Experimental GLP-1-oriented platforms have been developed using *Lactococcus lactis* [22,23,24,25,26,27,28,29], *Lactobacillus gasseri* [30,31], *Lacticaseibacillus paracasei* [32,33,34,35], and *Lactiplantibacillus plantarum* [36,37,38]. Additional platforms have employed *Bifidobacterium longum* [39], *Escherichia coli* Nissle 1917 (EcN) [40,41,42,43], *Clostridium butyricum* [44], *Saccharomyces cerevisiae* [45,46,47], and *Saccharomyces boulardii* [48]. These systems encompass three principal strategies: production of GLP-1 or minimally modified GLP-1-derived peptides, production of modified GLP-1RAs or fusion proteins, and secretion of microbial effectors that stimulate endogenous GLP-1 release. Early investigations demonstrated recombinant peptide production and evaluated biological activity in experimental systems. Subsequent studies examined peptide modifications, protective formulations, and regulated expression systems.

Terminology in this field is not uniform. The term “engineered probiotics” is commonly applied to genetically modified microorganisms developed for defined biological functions [49], whereas “next-generation probiotics” may refer to microorganisms selected on the basis of their beneficial properties without genetic modification [50,51,52]. In regulatory terminology, a live biotherapeutic product contains viable microorganisms intended for the prevention, treatment, or cure of disease and is not necessarily genetically modified [53]. In this review, “living therapeutics” refers to genetically engineered viable microorganisms developed to produce a defined therapeutic effect.

A central challenge is determining whether a microbially produced molecule reaches an anatomical compartment containing its intended molecular target. Detection of a recombinant peptide in microbial culture medium does not establish its stability in the gastrointestinal lumen, diffusion through mucus, access to the epithelial surface, transport across the intestinal epithelium, or systemic exposure [54,55,56,57]. Changes in glucose tolerance, body weight, or inflammatory markers alone likewise do not establish systemic delivery of intact recombinant peptide or direct GLP-1R activation. GLP-1R involvement has been demonstrated using receptor knockdown in intestinal epithelial cell cultures and genetic receptor deletion in animal models [23,30], whereas other studies indicate that biological activity associated with the microbial chassis may contribute to metabolic outcomes [34]. Stimulation of endogenous GLP-1 secretion represents a further mechanism that does not necessarily require systemic absorption of the recombinant microbial effector [58,59]. Distinguishing these mechanisms requires direct assessment of product exposure and receptor or pathway involvement.

This review critically examines the experimental evidence for genetically engineered microorganisms designed to produce GLP-1-related peptides, modified GLP-1RAs, or effectors that stimulate endogenous GLP-1 secretion for the treatment of obesity and T2DM. The analysis focuses on recombinant product characterization, intestinal and systemic exposure, receptor involvement, attribution of reported metabolic effects to the engineered constructs, and the principal limitations affecting clinical translation.

## 2. Literature Search Strategy

PubMed/MEDLINE, Europe PMC, and OpenAlex were searched for English-language publications from database inception through 9 October 2026. Search terms included combinations of “engineered microorganisms”, “engineered probiotics”, “living therapeutics”, “synthetic biology”, “microbial delivery”, “GLP-1”, “glucagon-like peptide-1”, “GLP-1 receptor agonist”, “incretin”, “diabetes”, “type 2 diabetes”, and “obesity”. Additional searches used microbial species and recombinant construct names, supplemented by reference-list screening. Eligible studies were peer-reviewed original articles describing genetically engineered microorganisms developed to produce GLP-1-related therapeutic molecules or stimulate endogenous GLP-1 secretion. Studies limited to recombinant expression or in vitro functional evaluation were included if the engineered microorganism was intended for therapeutic use. Studies of unmodified microorganisms, recombinant proteins without an engineered microbial platform, and interventions unrelated to GLP-1 were excluded. Reviews and other non-original publications were used for background information but excluded from the primary dataset. Studies conducted in other disease models were considered separately as mechanistic or translational comparators.

**Figure 1:**
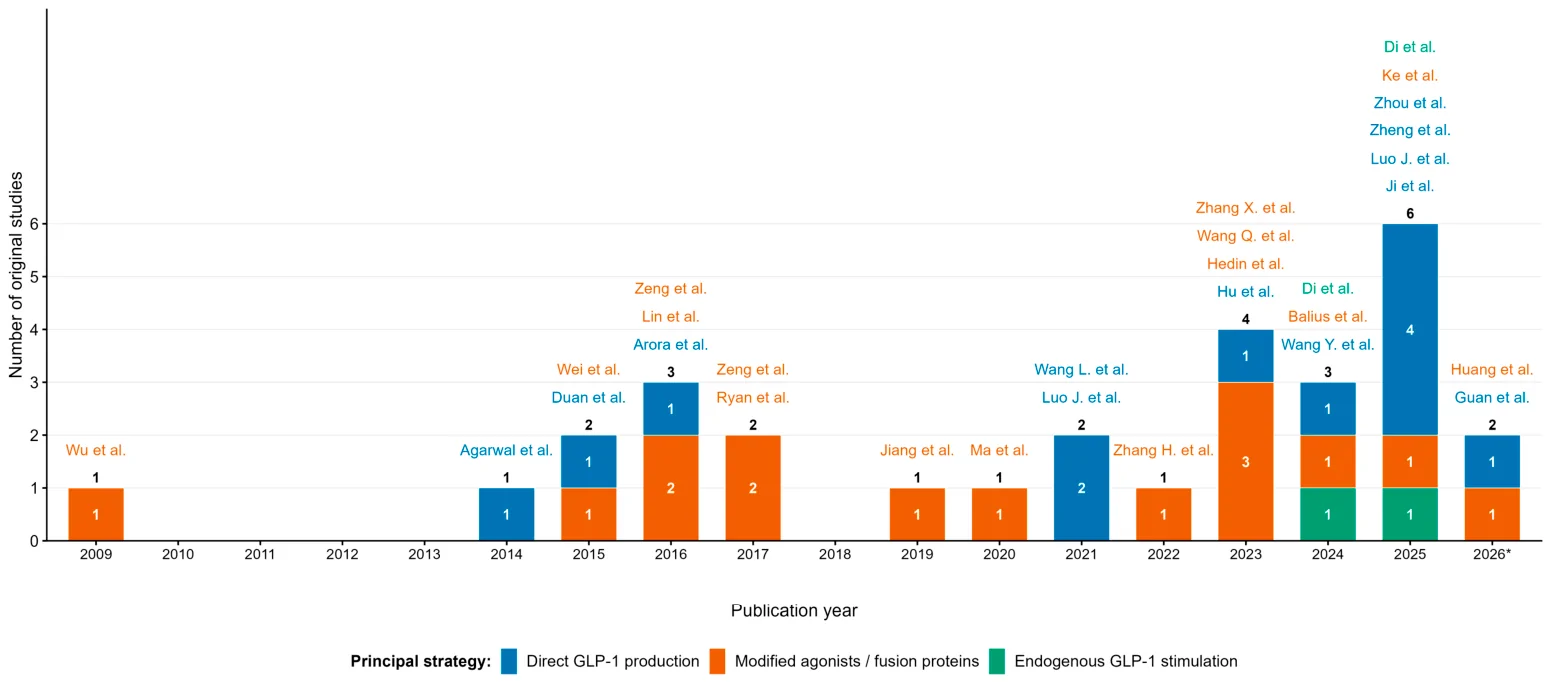
Temporal distribution of original studies on GLP-1-oriented engineered microbial therapeutics published between 2009 and 2026. Stacked bars indicate annual publication counts by principal experimental strategy: direct GLP-1 or GLP-1-derived peptide production, modified GLP-1RA or fusion protein production, and stimulation of endogenous GLP-1 secretion. Author labels identify individual studies, with reference numbers shown in square brackets [22,23,24,25,26,27,28,29,30,31,32,33,34,35,36,37,38,39,40,41,42,43,44,45,46,47,48,58,59]. Numbers above the bars indicate annual totals. * 2026 includes publications available through 9 October.

The final dataset comprised 29 original studies published between 2009 and 2026, classified according to their principal experimental strategy: direct GLP-1 or GLP-1-derived peptide production (*n* = 12), modified GLP-1RA or fusion protein production (*n* = 15), and stimulation of endogenous GLP-1 secretion (*n* = 2). Of these, 16 studies were published between 2022 and 2026. Figure 1 presents the annual distribution of the original studies, with each study counted once according to its publication year. The 2026 data include publications available through 9 October.

## 3. Metabolic and Intestinal Physiology Relevant to GLP-1-Oriented Living Therapeutics

### 3.1. Metabolic Dysfunction and Incretin Responses in Obesity and Type 2 Diabetes

Obesity-associated adipose tissue dysfunction contributes to insulin resistance through altered adipokine secretion, increased release of non-esterified fatty acids, ectopic lipid deposition, and chronic low-grade inflammation [60,61,62,63,64]. In T2DM, progressive pancreatic β-cell dysfunction, inappropriate glucagon secretion, and impaired incretin action contribute to dysregulated glucose homeostasis [65,66,67,68,69].

The gastrointestinal tract regulates glucose metabolism through nutrient sensing, incretin secretion, bile acid signaling, microbial metabolites, and neural pathways [70,71]. Although the physiological incretin effect is attenuated in T2DM, responsiveness to pharmacological GLP-1R activation is largely preserved [14]. This distinction provides a physiological basis for investigating both direct delivery of GLP-1RAs and stimulation of endogenous GLP-1 secretion.

Diet, medication use, intestinal transit, inflammatory status, bile acid composition, and resident microbiota vary among individuals with obesity and T2DM [72,73,74,75,76]. These factors may affect survival and intestinal localization of orally administered microorganisms and recombinant product formation, although their influence on therapeutic efficacy is poorly characterized.

### 3.2. Microbial Metabolites and Endogenous GLP-1 Secretion

The gut microbiota contributes to host metabolism through the production and transformation of metabolites that influence epithelial, enteroendocrine, immune, and neural pathways, including those involved in endogenous GLP-1 secretion [77,78,79].

Short-chain fatty acids (SCFAs), including acetate, propionate, and butyrate, function as metabolic substrates and signaling molecules [77,79]. SCFAs activate free fatty acid receptors 2 and 3 (FFAR2 and FFAR3), which are expressed in enteroendocrine and other intestinal cells [79,80]. SCFAs can stimulate GLP-1 and peptide YY (PYY) secretion, with experimental evidence supporting the involvement of FFAR2 [80].

Microbial bile acid metabolism provides another link between the gut microbiota and enteroendocrine signaling. Through deconjugation, dehydroxylation, and epimerization, intestinal microorganisms modify the composition and signaling properties of the bile acid pool [81]. Bile acids act through farnesoid X receptor (FXR) and G protein-coupled bile acid receptor 1 (TGR5/GPBAR1). TGR5 activation in enteroendocrine L cells stimulates cAMP signaling and GLP-1 secretion, whereas FXR contributes to the regulation of bile acid and metabolic homeostasis [81].Enteroendocrine cells integrate nutrient- and microbiota-derived signals and communicate with local neural, immune, epithelial, and vascular compartments [82,83]. These pathways are relevant to engineered microorganisms designed to stimulate endogenous GLP-1 secretion. However, the contribution of individual microbial effectors and their access to the relevant host targets require experimental verification.

### 3.3. Intestinal Barrier Function and Host–Microbe Interactions

The intestinal barrier comprises mucus, epithelial cells and junctional complexes, antimicrobial factors, immune populations, vasculature, lymphatics, and the resident microbiota [84,85]. These components determine the spatial relationship between an engineered microorganism, its encoded product, and potential host targets. Mucus properties, epithelial permeability, inflammation, and microbial competition may therefore influence microbial persistence, local product availability, and access to the mucosal surface.

Obesity and metabolic disease are associated with alterations in mucus properties, epithelial integrity, and translocation of microbial products, although the extent and clinical significance of these changes vary across experimental systems and human studies [84,85,86]. For living therapeutics, microbial localization and recombinant product distribution are more informative for assessing intestinal exposure than junctional protein expression alone.

**Figure 2:**
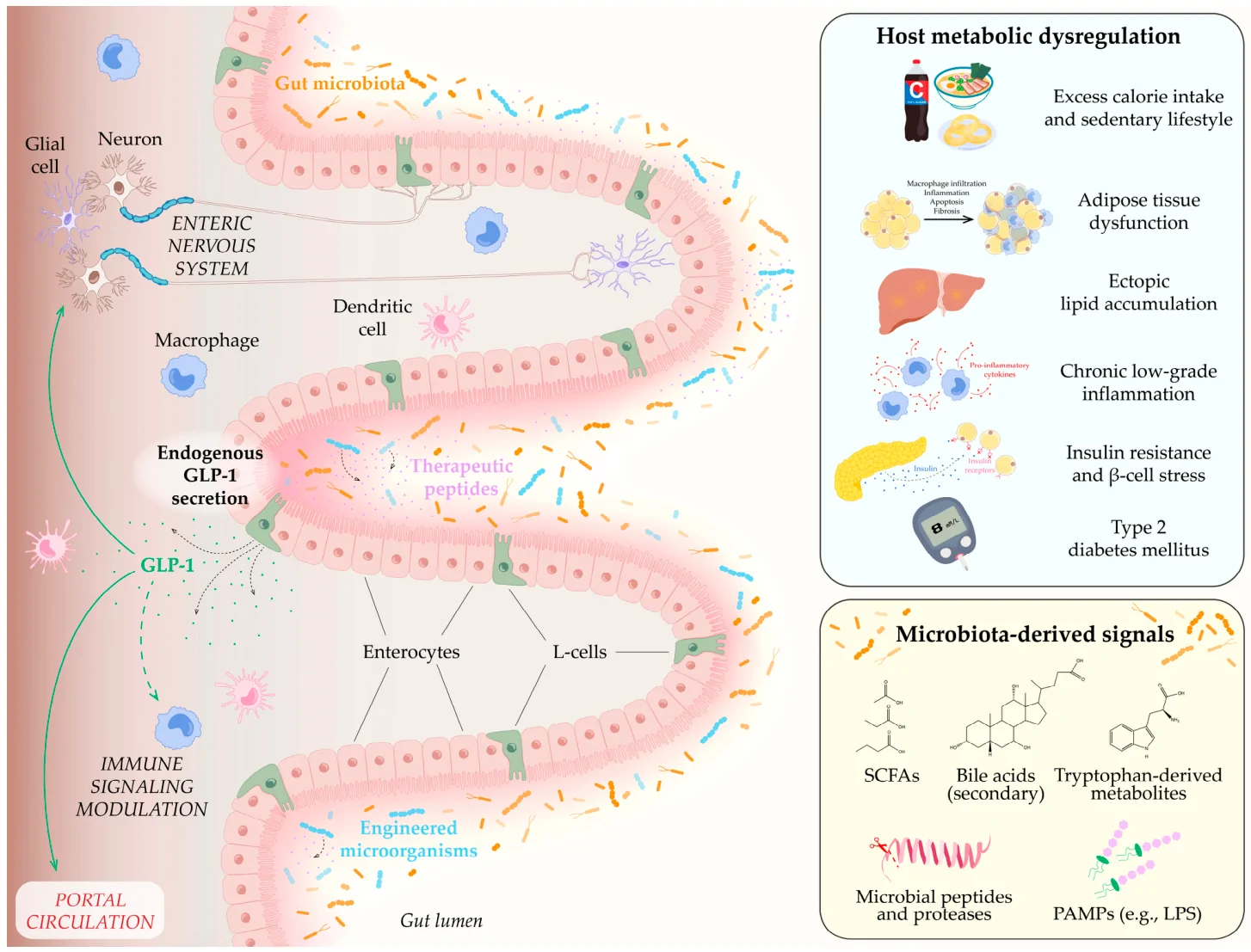
Bidirectional relationships among obesity, T2DM, the gut microbiota, and intestinal barrier function. Obesity-associated metabolic disturbances influence microbial composition, intestinal physiology, and barrier properties. Microbial metabolites and microbe-associated signals affect epithelial integrity, enteroendocrine signaling, immune activity, neural communication, and portal metabolism. Engineered living therapeutics may act at intestinal sites of interaction without requiring systemic exposure to the recombinant product. Abbreviations: GLP-1, glucagon-like peptide-1; SCFAs, short-chain fatty acids; PAMPs, pathogen-associated molecular patterns; LPS, lipopolysaccharide.

Engineered microorganisms may influence intestinal barrier function through metabolites, secreted proteins, and interactions with mucus or resident microorganisms [77,79,84,85]. These effects may contribute to treatment-associated phenotypes but must be distinguished experimentally from direct GLP-1- or GLP-1R-mediated mechanisms. Figure 2 summarizes these relationships and potential sites of local action of engineered living therapeutics.

## 4. GLP-1 Physiology and Intestinal Mechanisms of Engineered Microbial Therapeutics

### 4.1. GLP-1 Physiology, Signaling, and Receptor Distribution

GLP-1 is generated through tissue-specific processing of proglucagon. Prohormone convertase 1/3 mediates GLP-1 production in intestinal enteroendocrine L cells, whereas prohormone convertase 2 predominantly generates glucagon in pancreatic α-cells [87,88,89]. The principal biologically active forms are GLP-1(7–36)amide, which predominates in human circulation, and GLP-1(7–37) [87]. The *N*-terminally extended GLP-1(1–37), expressed by several engineered microbial platforms, has substantially lower insulinotropic activity than mature GLP-1(7–37) [87,89]. Native GLP-1 undergoes rapid *N*-terminal cleavage by DPP-4, with most of the secreted peptide inactivated before reaching the systemic circulation [87]. Its plasma half-life is approximately 1–2 min [87,88,90].

GLP-1-producing enteroendocrine L cells are distributed throughout the intestine, with secretion regulated by carbohydrates, lipids, amino acids, bile acids, SCFAs, and neural and paracrine signals [83,87,91]. GLP-1 is released from L cells into the intestinal interstitium and circulation through basolateral secretion [87,91], although Grau-Bové et al. reported predominantly apical secretion in ex vivo preparations of human colonic mucosa [92].

GLP-1R is a class B1 G protein-coupled receptor that signals predominantly through Gs-mediated activation of adenylyl cyclase and accumulation of cAMP [90,93]. In pancreatic β-cells, cAMP activates protein kinase A and exchange protein directly activated by cAMP 2, potentiating glucose-dependent insulin secretion [90,93]. GLP-1R activation also suppresses glucagon secretion through intra-islet mechanisms, delays gastric emptying, and reduces food intake through peripheral and central neural pathways [87,88,90].

Local GLP-1 signaling may involve intestinal and neural pathways [87,94]. In male mice, Cao et al. identified apparent contacts between vagal axons and fewer than 0.5% of GLP-1-producing cells in the ileum and colon, supporting predominantly paracrine rather than direct synaptic communication [95].

GLP-1R localization has been investigated using immunohistochemistry and ligand binding approaches. Pyke et al. detected GLP-1R in pancreatic β-cells and, within human and nonhuman primate gastrointestinal tissues, in gastric parietal cells, duodenal Brunner’s glands, and neurons of the myenteric plexus [96]. More recently, Dontamsetti et al. reported GLP-1R immunoreactivity in mucosal nerve fibers, submucosal and myenteric plexuses, and enteric neuronal populations of the human gastrointestinal tract, with greater expression in distal intestinal regions [97]. Grau-Bové et al. also reported GLP-1R immunostaining in the human colonic epithelium [92], although functional receptor accessibility from the intestinal lumen remains uncharacterized.

### 4.2. Intestinal Exposure and Target Accessibility of Microbial Products

Regions of the gastrointestinal tract differ in pH, oxygen and nutrient availability, bile acid composition, protease activity, mucus organization, transit time, and microbial density [56,98,99]. Direct sampling of the human intestine has demonstrated regional variation in microbial composition, host proteins, and bile acid profiles [98]. These conditions may influence the survival and metabolic activity of orally administered microorganisms [100,101].

Recombinant protein expression under in vitro culture conditions provides limited information about intestinal exposure following oral administration. The administered microbial dose, expressed in colony-forming units (CFU), does not indicate the amount of recombinant product released in vivo. Similarly, protein concentrations measured in culture supernatants reflect extracellular accumulation under defined conditions. In the intestine, product availability depends on microbial survival and localization, recombinant expression, release, degradation, and transit [57,100,101]. The release of recombinant proteins also depends on their cellular localization. Secreted proteins enter the extracellular environment, surface-displayed proteins remain associated with the microbial cell envelope, and intracellular proteins may be released following cell disruption or lysis [57,101].

Following luminal release, recombinant peptides encounter host and microbial proteases whose activities vary between intestinal regions [56,102,103]. Proteolysis may also occur at the epithelial surface, where DPP-4 is localized to the apical membrane of intestinal cells [104]. Susceptibility to degradation depends on peptide sequence and structure, including amino acid substitutions and fusion with additional protein domains [102,103].

Diffusion of peptides and proteins through intestinal mucus depends on molecular size, charge, hydrophobicity, mucus composition, and interactions with mucins [105]. The intestinal epithelium further restricts the passage of intact peptides into underlying tissues through limited transcellular transport and paracellular permeability regulated by intercellular junctions [56,102,103]. Following epithelial passage, absorbed molecules enter the lamina propria and may reach the mesenteric and portal venous circulation, with subsequent systemic distribution influenced by intestinal and hepatic metabolism.

The effects of peptide formulation and molecular modification on intestinal transport have been investigated in nonmicrobial delivery systems. Qu et al. compared native GLP-1 with GLP-1 incorporated into a silica nanomatrix using Caco-2 monolayers and everted rat intestinal sacs [106]. In the everted gut sac model, 24% of formulated GLP-1 crossed the intestinal mucosa over 180 min, compared with 5% of native GLP-1, whereas basolateral-to-apical transport in Caco-2 monolayers did not differ significantly between formulations. In Caco-2 monolayers, Kweon et al. reported apparent permeability coefficients for apical-to-basolateral transport of 0.04 ± 0.04 × 10^−6^ cm/s for exenatide and 3.52 ± 0.52 × 10^−6^ cm/s for bD-G1A, a modified GLP-1RA conjugated to two deoxycholic acid moieties [107].

Engineered microorganisms have also been investigated for modifying epithelial permeability and transporting recombinant protein cargo. Gelli et al. engineered *S. boulardii* to produce peptides that increased epithelial permeability in Caco-2 cultures and enhanced intestinal absorption of a model macromolecule in mice [108]. Gong et al. developed an engineered EcN platform that packages recombinant proteins into outer membrane vesicles, which crossed the intestinal epithelium and reached extraintestinal tissues following oral administration in mice [109]. These approaches may be relevant to the intestinal delivery of therapeutic peptides and proteins, although neither study investigated GLP-1RAs.

**Figure 3:**
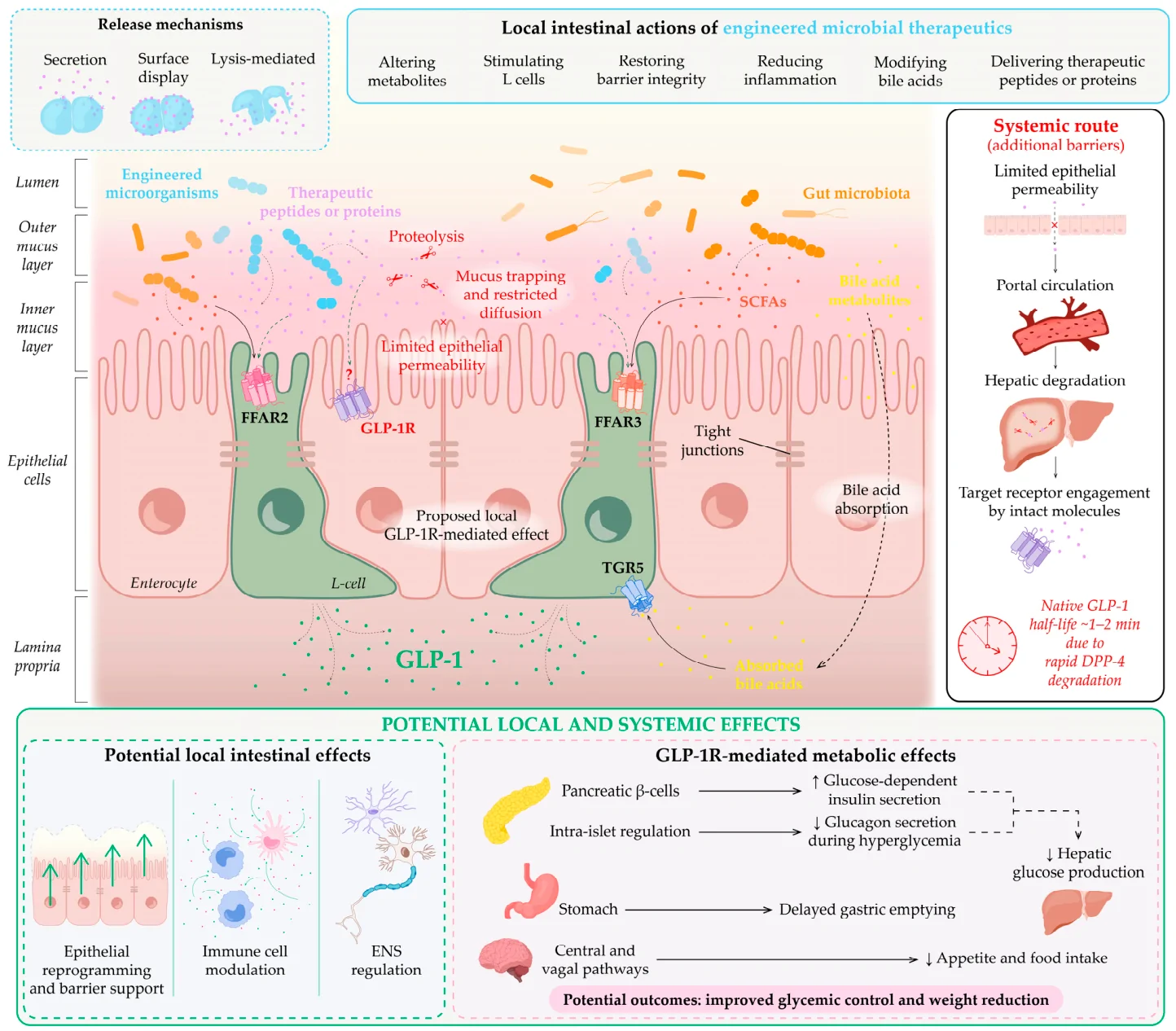
Intestinal target accessibility and potential local and systemic effects of engineered microbial therapeutics. Engineered microorganisms may secrete recombinant peptides or proteins, display them on the cell surface, or release them following cell lysis. Luminal products encounter proteolytic degradation, mucus retention, restricted diffusion, and limited epithelial permeability. Microbial SCFAs and bile acid metabolites may stimulate enteroendocrine signaling through FFAR2/FFAR3-associated pathways and TGR5/GPBAR1, respectively. Intestinal absorption of bile acids enables access to predominantly basolateral TGR5 and subsequent GLP-1 secretion. The membrane positions of FFAR2 and FFAR3 are schematic and do not imply established apical localization. The apical GLP-1R represents a hypothetical site of receptor activation, as functional GLP-1R accessibility at the luminal surface of intestinal epithelial cells remains unconfirmed. Abbreviations: GLP-1, glucagon-like peptide-1; GLP-1R, GLP-1 receptor; FFAR2/FFAR3, free fatty acid receptors 2 and 3; TGR5/GPBAR1, G protein-coupled bile acid receptor 1; DPP-4, dipeptidyl peptidase-4; SCFAs, short-chain fatty acids; ENS, enteric nervous system.

Microbial effectors may stimulate endogenous GLP-1 secretion locally without systemic absorption. Enteroendocrine L cells express receptors responsive to microbial metabolites, including FFAR2, FFAR3, and TGR5/GPBAR1 [80,81,91]. In colonic mucosa, Tough et al. observed FFAR2-associated responses after both apical and basolateral stimulation, whereas FFAR3 signaling involved enteric neurons [110]. Brighton et al. demonstrated that bile acids stimulate GLP-1 secretion predominantly through basolaterally accessible TGR5/GPBAR1 [111]. The intestinal barriers and potential routes of recombinant product exposure are illustrated in Figure 3.

### 4.3. Interpretation of Local and Systemic Effects

The anatomical site of GLP-1R activation and the molecular origin of the activating ligand represent distinct questions in studies of engineered microorganisms. Duan et al. reported the appearance of insulin-producing intestinal epithelial cells and improved glucose tolerance in diabetic rats receiving GLP-1(1–37)-producing *L. gasseri*, without a significant increase in circulating GLP-1 [30]. GLP-1R knockdown prevented epithelial reprogramming in cultured cells, although receptor dependence was not examined in the treated animals. In contrast, Arora et al. reported increased portal GLP-1 concentrations and improved glucose tolerance following administration of recombinant *L. lactis* [23]. The metabolic response was absent in GLP-1R-deficient mice, but the measured GLP-1 could not be attributed specifically to the recombinant product.

Interpretation of GLP-1 measurements depends on the molecular forms detected. Immunoassays differ in their recognition of intact GLP-1 and its metabolites [112], whereas immunoaffinity LC–MS/MS allows separate quantification of GLP-1(7–36)amide and GLP-1(9–36)amide in human plasma [113]. When recombinant and endogenous GLP-1 share identical sequences, their molecular origin cannot be distinguished by sequence analysis alone.

Recombinant GLP-1RAs may exert local effects within intestinal tissues without substantial systemic exposure, whereas increased portal GLP-1 concentrations may reflect absorption of microbial peptides, stimulation of endogenous GLP-1 secretion, or both [23,30]. These mechanisms may operate concurrently, complicating attribution of GLP-1R-dependent metabolic effects to the recombinant product.

## 5. GLP-1-Oriented Living Therapeutics: Preclinical Evidence

Preclinical studies of engineered microorganisms targeting GLP-1 can be grouped according to the identity and origin of the biologically active product. The first group comprises strains producing GLP-1 or GLP-1-derived peptides. The second comprises modified GLP-1RAs and fusion proteins designed to alter peptide stability, uptake, or persistence. The third uses microbial products to stimulate endogenous GLP-1 secretion. These approaches differ in their requirements for peptide processing, intestinal transport, and receptor activation.

### 5.1. Direct Production of GLP-1 and GLP-1-Derived Peptides

Early studies demonstrated the feasibility of engineering lactic acid bacteria to produce GLP-1 peptides and influence glucose regulation in experimental models. Agarwal et al. engineered *L. lactis* to secrete a recombinant peptide containing the human GLP-1(7–36) sequence and examined its biological activity in HIT-T15 cells, epithelial transport in MDCK monolayers, and metabolic effects in Zucker diabetic fatty rats [22]. The secreted product stimulated glucose-dependent insulin secretion in vitro, while oral administration of the engineered strain was associated with a transient reduction in blood glucose. However, the construct contained additional *N*-terminal residues, and processing to the mature peptide was not characterized. The molecular identity and intestinal or systemic exposure of the recombinant product after bacterial administration were not determined.

Duan et al. investigated *L. gasseri* expressing GLP-1(1–37), the full-length form that differs from mature incretin GLP-1, in rats with streptozotocin-induced diabetes [30]. Treatment improved glucose tolerance and was associated with the appearance of insulin-positive intestinal epithelial cells expressing pancreatic β-cell markers. GLP-1 immunoreactivity was detected at the luminal surface of the upper intestine without a corresponding increase in circulating GLP-1. GLP-1R knockdown using siRNA prevented GLP-1(1–37)-induced epithelial reprogramming in cultured IEC-6 cells, whereas receptor dependence of the intestinal and metabolic responses was not examined in treated rats. However, immunofluorescence did not establish the molecular identity of the detected peptide, and the lineage origin and persistence of the induced insulin-positive cells after treatment discontinuation were not determined.

Arora et al. examined *L. lactis* producing murine GLP-1(1–37) and demonstrated increased insulin secretion in isolated pancreatic islets and improved glucose tolerance following oral bacterial administration [23]. The absence of these effects in mice lacking GLP-1R provided direct evidence of receptor dependence. Although portal GLP-1 concentrations increased after treatment, the assay did not distinguish recombinant peptide from endogenous hormone. The results therefore establish receptor involvement in the physiological response without directly demonstrating intestinal transport of the bacterial peptide.

Subsequent studies examined GLP-1-producing microorganisms in experimental models of obesity and T2DM. Wang et al. reported reduced weight gain, improved glucose tolerance, and decreased hepatic lipid accumulation following administration of recombinant *L. lactis* to mice fed a high-fat diet (HFD) [25]. The inclusion of an empty-vector control supported a contribution from the GLP-1 expression construct, although the active peptide was not measured at intestinal or systemic sites. Luo et al. extended the investigation of recombinant *L. plantarum* pMG36e-GLP-1 to rhesus macaques with spontaneous T2DM [36]. Seven weeks of oral administration was associated with reduced fasting glucose and changes in fecal microbial composition and metabolite profiles. However, the small cohort, absence of a parallel bacterial control group, and lack of recombinant peptide measurements prevented direct attribution of these changes to GLP-1 production.

Hu et al. evaluated a related *L. plantarum* construct in diabetic mouse models and reported improved glycemic measures and pancreatic β-cell function, accompanied by changes in inflammatory, apoptotic, and hepatic metabolic markers [37]. Comparisons with the parental bacterial strain and exenatide provided relevant controls, but did not establish the contribution of GLP-1R activation to the observed metabolic responses. In a separate approach, Wang et al. examined lipid-coated GLP-1-producing EcN and reported improvements in glycemic measures and pancreatic histology [41]. The study also characterized bacterial distribution and changes in pancreatic immune cell populations. These observations did not establish the tissue distribution of the recombinant peptide or its direct involvement in the pancreatic response.

Chromosomal engineering has also been used to reduce reliance on plasmid-based expression. Luo et al. developed an EcN strain carrying a chromosomally integrated GLP-1 expression cassette without an antibiotic selection marker [42]. Oral administration was associated with reduced hyperglycemia, improved pancreatic β-cell-related measures, and lower inflammatory and apoptotic markers in mouse models of T1DM and T2DM. However, the parental strain also reduced glucose concentrations in the T1DM model, indicating that bacterial effects independent of the recombinant construct contributed to the observed response. The absence of direct measurements of recombinant GLP-1 exposure and receptor dependence prevented further separation of these mechanisms.

Chromosomal integration was also investigated by Zheng et al., who used the native CRISPR-Cas9 system of *L. paracasei* NMG-13 to obtain the recombinant strain A4-2 carrying a human GLP-1 sequence [35]. Genomic integration was confirmed by sequencing, GLP-1 transcripts were detected by RT-qPCR, and recombinant peptide fragments were identified in bacterial lysates by nanoLC–MS. Although the integrated sequence was retained during serial passage, GLP-1 transcript abundance decreased. The study established the feasibility of chromosomal engineering in this bacterial species but did not examine peptide secretion, biological activity, or therapeutic effects following bacterial administration.

Zhou et al. expressed GLP-1(1–37) in *C. butyricum* and reported improvements in glucose regulation, lipid metabolism, and measures of insulin sensitivity, together with reduced hepatic injury in diabetic mice [44]. Changes in GLP-1R expression and markers associated with adenylyl cyclase and PKA activity were also observed. However, these measurements did not establish receptor dependence. The intrinsic metabolic activity of *C. butyricum*, including butyrate production, represents an additional potential contributor to the observed effects.

Other studies introduced modifications intended to improve bacterial survival or regulate peptide production. Ji et al. evaluated recombinant *L. lactis* expressing GLP-1 and modified with a surface coating containing dopamine and β-glucan [28]. The formulation improved bacterial survival under simulated gastrointestinal conditions, while oral administration was associated with improved glucose and lipid measures in diabetic mice. Because the coating altered the properties of the microbial carrier, the contribution of recombinant GLP-1 could not be evaluated independently of changes in bacterial survival and intestinal retention.

Guan et al. developed a glucose-regulated GLP-1 expression system in EcN using the HexR regulatory circuit [43]. Regulation of recombinant expression by glucose was demonstrated experimentally, and GLP-1R activation was examined using reporter cells. Oral administration improved glycemic regulation in diabetic mice and nonhuman primates. These experiments provide evidence of regulated microbial peptide production and biological activity, although circulating GLP-1 measurements did not specifically identify the intact bacterial product. Receptor dependence of the metabolic response was not tested directly in treated animals.

### 5.2. Modified GLP-1R Agonists and Fusion Proteins

Modification of GLP-1 and fusion with additional peptide domains have been investigated to reduce proteolytic degradation, facilitate epithelial transport, or extend peptide persistence. These modifications affect the molecular properties of the expressed products and may introduce additional requirements for proteolytic processing before receptor activation.

Wei et al. engineered *B. longum* to produce human GLP-1(7–37) fused to penetratin through a furin cleavage site [39]. Penetratin was incorporated to facilitate intestinal absorption of GLP-1, while the furin cleavage site was intended to permit release of the intact peptide. The fusion protein demonstrated insulinotropic activity following furin treatment. Administration of bacterial culture supernatant into isolated rat intestinal loops was followed by increased plasma GLP-1 immunoreactivity, indicating absorption under the experimental conditions. However, these experiments did not evaluate oral administration of viable recombinant bacteria or establish systemic exposure to the intact fusion protein following bacterial treatment.

Exendin-4, a GLP-1RA originating from *Heloderma suspectum* with intrinsic resistance to DPP-4 cleavage, has also been produced in engineered lactic acid bacteria. Zeng et al. expressed exendin-4 in *L. paracasei* L14, reporting a concentration of 28.5 nmol/L in culture supernatant after 12 h, and demonstrated its insulinotropic activity in INS-1 cells [33]. To evaluate intestinal epithelial transport, the authors used polarized Caco-2 monolayers and found that transepithelial transport of exendin-4 secreted by the engineered bacteria was greater than that of the free peptide, without compromising monolayer integrity. These findings support the feasibility of bacterial delivery across an epithelial cell model but do not establish intestinal absorption following oral administration of the producing strain. In a separate study, Zeng et al. used a nisin-inducible expression system in *L. lactis* NZ9000, reporting exendin-4 concentrations of up to 249 nmol/L in culture supernatant and increased insulin secretion accompanied by changes in PI3K/AKT signaling markers in INS-1 cells [24].

Lin et al. engineered *L. paracasei* BL23 to express pentameric GLP-1 derivatives designed to resist DPP-4 degradation and permit controlled proteolytic processing [32]. The constructs incorporated modifications affecting susceptibility to DPP-4 or trypsin, together with cleavage sites between GLP-1 units. Processed products stimulated insulin secretion in vitro. However, oral administration to Goto–Kakizaki rats did not significantly improve glucose tolerance compared with the parental strain, despite reductions in nonfasting glucose at selected time points. Plasmid instability and the absence of direct measurements of intestinal peptide exposure limited interpretation of the metabolic results.

Ryan et al. evaluated *L. paracasei* NFBC 338 expressing KGLP-1, a GLP-1 analogue designed to resist DPP-4 degradation [34]. Treatment altered circulating lipid concentrations in rats, but did not significantly improve glucose tolerance relative to the isogenic control strain. In a separate mouse experiment, glycemic responses did not differ significantly between animals receiving the recombinant and control strains. Several metabolic changes were also associated with the parental bacterial strain, indicating that the observed effects could not be attributed exclusively to KGLP-1 production.

Ma et al. engineered EcN to produce GLP-1 GM, a modified GLP-1 analogue, and examined its metabolic effects in mice fed an HFD [40]. Treatment reduced food intake, weight gain, and adiposity and improved glucose tolerance, accompanied by changes in hepatic lipid metabolism. Altered hypothalamic neuropeptide expression was also reported, but these measurements did not establish the anatomical site of action of the recombinant peptide or its dependence on GLP-1R.

Zhang et al. examined a GLP-1 analogue expressed in *L. lactis*, in which alanine at position 8 was replaced with serine (A8S) [26]. This modification reduced degradation by DPP-4 in vitro, while administration of the recombinant strain improved glycemic measures and reduced fat mass in db/db mice. The recombinant peptide was not distinguished analytically from endogenous circulating GLP-1, and receptor dependence of the metabolic effects was not determined.

Wang et al. developed *L. plantarum* WCFS1 expressing a GLP-1 analogue containing modifications intended to reduce degradation by DPP-4 and trypsin [38]. The recombinant product detected in culture retained the secretion signal, and smaller products appeared after trypsin treatment. Oral administration improved glucose tolerance and measures of pancreatic β-cell function in db/db mice. However, processing of the expressed protein in the intestine and exposure to the active peptide were not characterized following bacterial administration.

Zhang et al. developed a separate approach using optically regulated expression in *L. lactis* [27]. Blue light induced GLP-1 production, while an additional construct encoded a GLP-1 fusion with an Fc domain intended to prolong peptide persistence. The platform was evaluated in rodent metabolic models and demonstrated external control of recombinant expression. However, the molecular forms and concentrations of GLP-1 present in intestinal tissue or blood following bacterial administration were not characterized. Regulation of expression by light therefore demonstrates control of microbial production rather than direct evidence of peptide exposure at a pharmacological target.

Earlier studies investigated recombinant *S. cerevisiae* strains expressing multimeric GLP-1 analogues. Wu et al. developed strain SG2 containing a chromosomally integrated construct encoding ten tandem GLP-1 analogue sequences [45]. Recombinant protein production reached approximately 0.84 mg/g packed cells. Oral administration of lyophilized SG2 at 5 g/kg was associated with a reduction in blood glucose in hyperglycemic rats. Jiang et al. subsequently developed *S. cerevisiae* LHY168 expressing ten tandem repeats of a modified GLP-1 sequence [47]. Recombinant protein production reached approximately 1.56 mg/g wet cell mass. Oral administration at 6 g/kg for 16 days reduced blood glucose in mice with diet- and streptozotocin-induced diabetes. However, neither study established intestinal release of an intact receptor-active peptide, portal or systemic exposure, or GLP-1R dependence of the observed metabolic effects. Viability of the administered yeast preparations was not quantitatively characterized.

Hedin et al. engineered *S. boulardii* to express exendin-4 and evaluated its biological activity in isolated pancreatic islets and mice with diet-induced obesity [48]. GLP-1R dependence of insulin secretion was demonstrated using islets from mice lacking GLP-1R. In animals, recombinant yeast administration reduced food intake and increased weight loss during concurrent cold exposure, whereas a significant effect was not observed at room temperature. The dependence of the metabolic response on temperature was established under the experimental conditions, but GLP-1R involvement was not directly tested in treated animals.

Ke et al. engineered *L. gasseri* to secrete a fusion protein GPA, comprising GLP-1, a protein transduction domain intended to facilitate intestinal absorption, and an ABP designed to prolong systemic circulation [31]. The additional domains were incorporated at the *C*-terminus of GLP-1 to preserve the *N*-terminal region required for insulinotropic activity. The secreted fusion protein stimulated insulin release in MIN6 cells, while oral administration of the engineered strain improved glycemic regulation and lipid metabolism in db/db mice. The authors also reported detection of GPA in rat blood by HPLC and immunoblotting following oral administration of the engineered strain. However, the individual contributions of the protein transduction and albumin binding domains to intestinal transport and systemic persistence were not directly established. Quantitative pharmacokinetics of the recombinant fusion protein were not determined.

Huang et al. evaluated recombinant *L. lactis* strains producing a modified GLP-1 analogue, designated LGLP, or exendin-4 [29]. Administration of the LGLP-producing strain improved glycemic measures and pancreatic islet morphology in db/db mice. Experiments with purified fluorescent peptides demonstrated uptake by intestinal epithelial cells and transport across intestinal mucosa. LC–MS analysis identified the LGLP-specific fragment HGEGTFTSD in peripheral plasma following oral administration of recombinant bacteria. This finding demonstrates that material derived from the expressed peptide reached the circulation, although the presence and biological activity of intact circulating LGLP were not established. The transport experiments with purified peptides and the analytical detection of a recombinant peptide fragment therefore provide complementary, but distinct, evidence of intestinal absorption.

### 5.3. Stimulation of Endogenous GLP-1 Secretion

A different strategy involves engineering microorganisms to produce proteins that stimulate GLP-1 secretion from host enteroendocrine cells. In this case, the recombinant microbial product may act locally within the intestine without requiring systemic absorption. The proposed mechanism instead depends on interaction with a host target capable of regulating endogenous GLP-1 release.

Di et al. engineered *L. lactis* NZP9 to secrete P9/Amuc_1631, a protein derived from *A. muciniphila*, using the Usp45 secretion signal [59]. Culture supernatant from the engineered strain increased GLP-1 secretion in NCI-H716 cells and was associated with increased expression of *GCG* and *PCSK1*. The study provided evidence of an enteroendocrine response to products released by the engineered strain but did not evaluate metabolic effects following bacterial administration or establish the molecular target responsible for the response. In a subsequent study, Di et al. engineered *L. lactis* P11P9 to secrete P9 and evaluated its metabolic effects in mice fed an HFD [58]. Recombinant P9 increased GLP-1 secretion in NCI-H716 cells, a human colorectal adenocarcinoma line used as an enteroendocrine model. In mice fed an HFD, bacterial administration reduced food intake, weight gain, and adipocyte hypertrophy and increased serum GLP-1 concentrations. Increased intestinal expression of *Gcg* and *Pcsk1* was also reported. Although P9 has been associated with interaction with ICAM-2, the requirement for this interaction in the response to the engineered strain was not directly established. The experiments therefore support stimulation of endogenous GLP-1 secretion without demonstrating the complete mechanism linking recombinant P9 to the metabolic effects.

In addition to proteins such as P9, short peptides of bacterial origin may also be expressed by engineered microorganisms to stimulate endogenous GLP-1 secretion. Brockmann et al. investigated this approach by engineering EcN to secrete HldSE, a phenol-soluble modulin peptide originating from *Staphylococcus epidermidis*, using an NSP4 secretion signal [114]. Administration of the recombinant strain increased circulating GLP-1, while pharmacological blockade of GLP-1R attenuated the protective effects of bacterial treatment. Although these experiments were performed in a model of colitis rather than diabetes or obesity, they demonstrate the feasibility of recombinant HldSE production and provide functional evidence of GLP-1R involvement in the host response. The metabolic effects of this platform have not been evaluated.

These studies address different aspects of endogenous GLP-1 stimulation. P9 production was associated with increased host GLP-1 secretion and metabolic responses in obese mice, whereas HldSE production was linked to pharmacologically demonstrated GLP-1R involvement in a distinct experimental setting. The findings establish biological activity of the expressed products without demonstrating that either mechanism has equivalent effects across different disease models.

### 5.4. Interpretation of Preclinical Findings

The available preclinical studies provide different levels of evidence regarding exposure to the recombinant product, involvement of GLP-1R, and the contribution of recombinant peptide production to metabolic effects. Detection of peptide fragments specific to the recombinant construct indicates the presence of material originating from the expressed product, but does not confirm the presence of an intact, biologically active peptide. Genetic deletion or pharmacological inhibition of GLP-1R can demonstrate receptor involvement in a physiological response but cannot independently identify the activating ligand or its route of transport.

Attribution of metabolic effects to the recombinant product also depends on the biological activity of the microbial strain. In several studies, parental or isogenic control strains produced metabolic changes, indicating that the observed responses were not attributable exclusively to the encoded product. Surface modifications and regulatory circuits introduce additional factors affecting bacterial survival, intestinal retention, or recombinant expression. Differences in peptide exposure, receptor dependence, and the responses of control strains affect how confidently the observed metabolic effects can be attributed to recombinant products (Table 1).

## 6. Engineering and Translational Development of GLP-1-Oriented Living Therapeutics

### 6.1. Chassis Selection and Genetic Architecture

The microbial chassis influences gastrointestinal survival, intestinal persistence, genetic tractability, recombinant protein processing, and host responses independent of the encoded therapeutic product. EcN has been investigated extensively as a chassis for therapeutic sensing, metabolic conversion, and recombinant product delivery [115,116,117]. *L. lactis* and lactobacilli possess established genetic tools for heterologous protein expression, although gastrointestinal survival, secretion capacity, and persistence vary among species and strains [118,119]. The spore-forming anaerobe *C. butyricum* has distinct survival characteristics, while its endogenous butyrate production may contribute to the metabolic responses observed in studies of GLP-1-expressing strains [44]. The yeast *S. boulardii* supports eukaryotic protein processing and secretion [120], although reported cases of *Saccharomyces* fungemia raise safety concerns in susceptible patients [121].

Genetic architecture affects recombinant expression and its stability during microbial propagation. Plasmid-based systems permit variation in gene copy number but are susceptible to segregational instability and may require selective maintenance. Lin et al. reported plasmid loss in recombinant *L. paracasei* strains expressing pentameric GLP-1 derivatives [32]. Chromosomal integration reduces dependence on plasmid maintenance, although expression remains influenced by integration site and genetic context [122]. Balius et al. confirmed chromosomal integration of a galactose-inducible exendin-4 fusion construct in *S. cerevisiae* and detected the recombinant protein following cell disruption [46]. Zheng et al. demonstrated retention of an integrated GLP-1 sequence in *L. paracasei* after 50 serial passages, accompanied by decreased GLP-1 transcript abundance [35]. These observations distinguish genomic integration and sequence retention from sustained recombinant expression.

Additional approaches to genomic engineering have been evaluated in microbial therapeutics for other indications. Triassi et al. used transcriptionally insulated chromosomal integration sites and regulated pathway expression to reduce metabolic burden in an engineered EcN platform for phenylketonuria [123]. Adolfsen et al. improved bacterial phenylalanine conversion through enzyme engineering [124]. These studies provide experimental evidence for the effects of genomic organization and pathway design on the functional activity of engineered microorganisms, although neither approach was evaluated for GLP-1 production.

### 6.2. Expression Control and Product Release

Constitutive promoters drive transgene expression without an external inducer, although recombinant product formation remains dependent on microbial growth and physiological conditions. Regulated systems provide control through external or metabolic inputs. Zhang et al. developed a light-responsive *L. lactis* platform producing GLP-1 and an Fc fusion variant, with metabolic effects evaluated in rodent models [27]. Guan et al. engineered a HexR-based glucose-responsive expression system in EcN and reported regulated GLP-1 production and glycemic effects in diabetic mice and nonhuman primates [43]. Both studies demonstrate regulated recombinant expression in GLP-1-oriented microbial platforms.

Regulation of transgene expression does not necessarily establish proportional control of pharmacological activity. The HexR circuit responds to glucose available to the microorganism, which may differ from systemic glucose concentrations. The quantitative relationship between local glucose availability, recombinant peptide production, and pharmacodynamic responses remains incompletely characterized [43]. Optical regulation likewise depends on the delivery of an external light stimulus to the engineered bacteria [27].

Product release introduces additional constraints. Secreted peptides may undergo extracellular processing and proteolytic degradation, making the molecular form reaching the intended host target an important determinant of biological activity [54,55,57]. Surface-associated presentation provides an alternative mode of interaction that depends on accessibility of the host target [118]. Intracellular products may also be released through programmed bacterial lysis. Synchronized lysis has been demonstrated in engineered bacterial systems developed for other therapeutic applications [125], but has not been evaluated as a GLP-1 delivery mechanism.

### 6.3. Persistence, Biocontainment, and Biological Safety

Intestinal persistence determines the duration of microbial residence but does not independently establish the period of recombinant product formation. Microbial recovery from feces provides information on shedding and clearance without defining the spatial distribution or functional activity of microorganisms within the intestine [98,115,126]. These characteristics require separate evaluation when interpreting the relationship between administered microorganisms and therapeutic activity.

Clinical investigation of engineered EcN has provided evidence that persistent colonization is not required for measurable pharmacodynamic activity. In a phase I/IIa study of SYNB1618, an engineered strain designed to metabolize intestinal phenylalanine, Puurunen et al. reported dose-dependent increases in phenylalanine-derived metabolites in plasma and urine. The administered strain was no longer detected in fecal samples within four days after treatment cessation [126]. These findings establish microbial metabolic activity during administration and subsequent fecal clearance, but do not demonstrate clinical efficacy for phenylketonuria.

Biological containment has been investigated using nutrient dependence and genetically encoded elimination systems. Steidler et al. replaced the *thyA* gene of *L. lactis* with a human IL-10 expression cassette, generating a thymidine-dependent strain [127]. In a subsequent phase I study involving patients with Crohn’s disease, Braat et al. reported retention of thymidine dependence and IL-10 production in bacteria recovered from fecal samples [128]. Synthetic auxotrophy based on dependence on noncanonical amino acids has also been demonstrated in genetically modified *E. coli* [129,130]. These containment strategies have not been experimentally validated in GLP-1-producing therapeutic strains.

Conditional elimination provides a separate approach to controlling microbial viability. The Deadman and Passcode circuits enable bacterial elimination in response to defined environmental inputs [131]. Rottinghaus et al. developed CRISPR-based kill switches in EcN and demonstrated bacterial elimination in the mouse intestine and temperature-dependent killing following excretion [132]. Containment efficiency can vary with environmental conditions. Hartig et al. reported escape frequencies approximately three to four orders of magnitude higher in natural surface waters than in nutrient-rich laboratory medium for bacteria carrying a CRISPR-based kill switch [133].

Biological safety also depends on microbial characteristics and host susceptibility. Potential hazards include translocation across the intestinal barrier, extraintestinal infection, inflammatory responses, and antimicrobial resistance [53,121]. The recombinant therapeutic product requires separate assessment. Modified GLP-1 sequences and fusion domains have been incorporated into several microbial platforms, but their immunogenicity after repeated bacterial administration has not been systematically characterized [27,31,39,48].

Environmental risk assessment additionally encompasses shedding of viable microorganisms, survival outside the host, and potential transfer of recombinant genetic material. The effectiveness of containment and decontamination measures under relevant environmental conditions remains an important consideration [133,134].

### 6.4. Manufacturing, Formulation, and Potency

Manufacturing conditions influence microbial viability, genetic stability, and functional activity. Cell banking, fermentation, harvesting, formulation, and storage can affect product consistency [135,136]. Quality characterization includes microbial identity, purity, viable-cell concentration, genetic stability, and biological potency [53,135,136]. However, viable-cell enumeration does not establish the amount or activity of the recombinant therapeutic product. For GLP-1-oriented platforms, potency assessment depends on the intended mechanism and may involve GLP-1R activation or stimulation of endogenous GLP-1 secretion. Modified agonists and fusion proteins require additional characterization of molecular identity, processing, and biological activity.

Formulation affects microbial survival during gastrointestinal transit and the physiological state of cells reaching the intended intestinal site. Protective coatings and encapsulation have been investigated to improve delivery of viable microorganisms [100,101]. Spore-forming bacterial chassis provide an alternative with distinct survival properties [44]. Differences in survival and intestinal residence may affect recombinant product formation after administration, making administered CFU and functional pharmacological output separate measures of dose.

### 6.5. Translational Validation and Experimental Models

Microbial culture studies permit characterization of recombinant expression, product formation, and genetic stability, whereas mammalian cell assays provide information on biological activity, receptor signaling, and cellular responses. These systems do not reproduce the organization and physiological conditions of the gastrointestinal tract. Polarized epithelial cultures, excised intestinal tissues, and intestinal microphysiological systems provide additional approaches for investigating epithelial transport, host–microbial interactions, and product distribution [29,105,137,138].

Experimental and computational models have been used to characterize the intestinal activity of engineered microorganisms developed for other indications. Nelson et al. investigated phenylalanine metabolism by engineered EcN in a human gut-on-a-chip system, including substrate consumption, metabolite formation, and transport between intestinal and vascular compartments [138]. Charbonneau et al. developed a mechanistic model incorporating bacterial metabolic activity and gastrointestinal physiological parameters to predict SYNB1618 activity and its potential effects on circulating phenylalanine concentrations [139]. These approaches address distinct experimental and quantitative aspects of microbial therapeutic activity.

For GLP-1-oriented platforms, validation requirements differ according to the intended mechanism. Systems producing GLP-1 or GLP-1RAs require characterization of recombinant product identity, processing, biological activity, and exposure in compartments relevant to receptor activation. Where systemic delivery is intended, intestinal epithelial transport represents an additional endpoint. Gleeson et al. reported differences in peptide permeability between Caco-2 Transwell cultures and a microfluidic gut-on-a-chip model using purified peptide formulations [137]. This investigation did not involve engineered microorganisms, but demonstrates that estimates of peptide permeability may depend on the experimental model. By contrast, platforms stimulating endogenous GLP-1 secretion require evidence linking the recombinant microbial effector to enteroendocrine responses, including involvement of the proposed upstream target and downstream signaling pathway. Appropriate product-negative and parental-strain controls are necessary to distinguish construct-dependent effects from those of the microbial chassis.

Clinical evaluation extends these experimental requirements to microbial persistence, clearance, shedding, safety, and pharmacodynamic activity [53,126]. Pharmacodynamic endpoints should reflect the intended mechanism, whether delivery of a recombinant GLP-1RA or stimulation of endogenous GLP-1 secretion.

### 6.6. Regulatory Considerations and Future Directions

Regulatory evaluation of living therapeutics depends on the identity and properties of the microbial strain, genetic modification, manufacturing process, and intended clinical application. FDA guidance concerning early clinical trials with live biotherapeutic products addresses chemistry, manufacturing, and control information, including product identity, purity, potency, and stability [53]. Additional considerations regarding analytical characterization, manufacturing consistency, and regulatory development have been described for living therapeutic products [135,136]. Requirements applicable to an individual genetically modified microbial product depend on its regulatory classification.

Genetic technologies investigated in other microbial applications provide additional approaches that have not been validated for GLP-1-oriented therapy. Brödel et al. demonstrated targeted base editing of intestinal *E. coli* in mice using non-replicative phage-derived delivery particles [140]. Unlike administration of a pre-engineered strain, this strategy modifies bacteria already present in the intestine. Its application to GLP-1-related therapeutic mechanisms has not been investigated. Multicomponent microbial constructs and more complex regulatory circuits would also require characterization of the functional contribution and stability of their individual genetic components [20,49].

Host-associated variability represents an additional consideration for clinical development. Interindividual differences in intestinal colonization by administered microorganisms have been demonstrated for unmodified probiotic strains [141]. Comparable relationships between host characteristics, persistence of engineered microorganisms, recombinant product formation, and pharmacodynamic activity have not been established for GLP-1-oriented living therapeutics. The predictive value of host or microbiome characteristics for patient stratification therefore remains unvalidated.

The available studies demonstrate the feasibility of microbial production of GLP-1-related therapeutic molecules, regulated expression, and several engineering approaches relevant to functional stability and containment. Clinical translation requires product-specific characterization of biological potency, persistence and clearance, safety, and pharmacodynamic activity. The principal translational constraints and corresponding experimental validation requirements are summarized in Table 2.

## 7. Conclusions

GLP-1-oriented living therapeutics encompass three distinct strategies: microbial production of GLP-1 or GLP-1-derived peptides, production of modified GLP-1RAs or fusion proteins, and stimulation of endogenous GLP-1 secretion. Studies in animal models have reported improvements in glycemic measures and, in some cases, reductions in body weight or weight gain. However, recombinant product expression and treatment-associated effects are generally better documented than exposure to intact biologically active products in vivo, receptor or pathway dependence, and the contribution of the engineered product relative to the microbial chassis.

These findings support further investigation of local intestinal mechanisms alongside systemic delivery of recombinant peptides. Clinical translation requires quantitative characterization of functional dose and biological potency, evidence linking recombinant product activity to a defined host response, genetic and functional stability, controlled microbial persistence, and appropriate biocontainment. No GLP-1-oriented engineered microbial therapeutic has yet demonstrated efficacy in humans, and clinical development remains dependent on reproducible manufacturing and acceptable biological and environmental safety.

## Figures and Tables

**Table 1:** Preclinical evidence for GLP-1-oriented engineered microbial therapeutics.

Microbial Platform and Genetic Construct	Encoded Product, Sequence Origin and Relevant Properties	Experimental Model and Study Design	Recombinant Product in Culture	Reported Biological or Metabolic Effects	Recombinant Product Detected In Vivo	Portal or Systemic Measurements	Receptor or Pathway Evidence	Principal Limitations	References
Direct GLP-1 production									
*Lactococcus lactis* LL-pUBGLP-1 (pUBGLP-1 plasmid)	GLP-1(7–36) sequence from human proglucagon, expressed with the Usp45 secretion signal and additional *N*-terminal residues. Processing to the mature peptide was not examined	HIT-T15 cells, MDCK monolayers, Zucker diabetic fatty rats	Approximately 60 pM recombinant GLP-1 after 12 h in 500 mL of culture medium (initial inoculum, 10^4^ CFU/mL; ELISA)	Glucose-dependent insulin secretion in HIT-T15 cells. After oral administration, blood glucose decreased by 10–20% between 2 and 11 h, and insulin AUC_0–11_ h was 2.5 times that of the group receiving a free GLP-1 solution	The intact recombinant peptide was not measured in intestinal tissue after oral bacterial administration	Transport was examined in MDCK monolayers. Portal and systemic concentrations of recombinant GLP-1 were not measured after oral administration of the engineered bacteria	Insulin secretion was measured in HIT-T15 cells. Dependence on GLP-1R was not tested	The additional *N*-terminal residues may affect peptide processing. Intestinal exposure to the recombinant molecule and GLP-1R dependence were not examined	[22]
*Lactobacillus gasseri* LG	GLP-1(1–37), the full-length form of GLP-1, expressed from a chromosomally integrated secretion cassette. The peptide was investigated for its ability to induce an insulin-producing phenotype in intestinal epithelial cells	IEC-6 cells and rats with diabetes induced by STZ	Production of GLP-1 was reported without a quantitative concentration in culture medium	Improved glucose tolerance and increased numbers of insulin-positive intestinal epithelial cells	GLP-1 immunoreactivity was detected along the luminal surface of the upper intestine by immunofluorescence. The concentration of intact recombinant peptide was not measured	Circulating GLP-1 did not increase after treatment	GLP-1R knockdown by siRNA inhibited epithelial reprogramming in IEC-6 cells. Receptor dependence was not tested in treated rats	The cellular origin and long-term stability of the induced insulin-positive phenotype were not established	[30]
*Lactococcus lactis* FI10937 (FI5876/pUK200_GLP1)	GLP-1(1–37) sequence from mouse proglucagon, fused to the Usp45 secretion signal	Isolated pancreatic islets and wild-type mice or mice lacking GLP-1R	Approximately 130 pg/mL active GLP-1 in culture supernatant	Enhanced insulin secretion in isolated islets and improved glucose tolerance in wild-type mice. The effects were absent in mice lacking GLP-1R	Portal GLP-1 was detected, but the assay did not distinguish recombinant peptide from endogenous GLP-1	Portal GLP-1 increased after oral administration. The molecular origin of the measured peptide was not determined	Experiments with islets and mice lacking GLP-1R demonstrated receptor dependence of the measured effects	The portal measurements did not distinguish bacterial GLP-1 from endogenous GLP-1 or identify the route of epithelial transport	[23]
*Lactiplantibacillus plantarum* (pMG36e-GLP-1)	GLP-1(1–37). The source species was not specified	Three rhesus macaques with spontaneous T2DM, assessed before and after treatment	GLP-1 expression was reported without a quantitative concentration in culture medium	Fasting glucose decreased from 7.33 ± 0.26 to 4.00 ± 0.29 mmol/L over seven weeks. The fecal microbiota and metabolite profiles also changed	Intestinal recombinant GLP-1 was not measured	Portal and systemic recombinant GLP-1 were not measured	No experiment directly tested GLP-1R involvement	The study included three animals and no parallel control group receiving the parental strain. Recombinant peptide exposure and receptor dependence were not examined	[36]
*Lactococcus lactis* MG1363 (pMG36e-GLP-1)	Full-length GLP-1(1–37)	Mice fed an HFD	Approximately 76 pg/mL GLP-1 in culture medium, measured by ELISA	Lower body weight gain, fasting glucose, serum triglycerides and hepatic lipid accumulation, with improved glucose tolerance. Insulin tolerance did not differ significantly between groups	Intestinal recombinant GLP-1 was not quantified	No portal or systemic assay distinguished bacterial GLP-1 from endogenous hormone	Receptor inhibition or genetic deletion was not used to test GLP-1R dependence	The empty vector control supports a contribution from the GLP-1 construct, but the concentration of active peptide at intestinal or systemic targets was not measured	[25]
*Lactiplantibacillus plantarum* (pMG36e-GLP-1)	GLP-1(1–37). Conversion to the mature active peptide was not characterized	Mice with diabetes induced by HFD and STZ, and db/db mice	GLP-1 expression was detected without a quantitative concentration of secreted peptide	Improved glycemic measures and indices of pancreatic β-cell function, with changes in pancreatic inflammatory and apoptotic markers and hepatic metabolic pathways	Intact bacterial GLP-1 was not measured in tissues	The recombinant peptide was not measured in portal or systemic blood	GLP-1R dependence was not examined by direct intervention	Comparisons included parental bacteria and exenatide, but the tissue concentration of recombinant GLP-1 was not measured	[37]
*Escherichia coli* Nissle 1917 (LEG, with a lipid surface coating)	Recombinant GLP-1. The sequence origin, amino acid boundaries and processing of the peptide were not specified	Diabetic mice and analyses of bacterial distribution in tissues	Expression of GLP-1 was reported without a quantitative measurement of recombinant peptide output	Improved glycemic measures and reduced injury to pancreatic islets, accompanied by changes in pancreatic immune cell populations	Bacterial distribution was examined, but the localization of intact recombinant GLP-1 was not measured	Recombinant GLP-1 in portal or systemic blood was not quantified	GLP-1R involvement was not tested by receptor inhibition or genetic deletion	The distribution of the bacteria and associated pancreatic changes do not establish the tissue distribution of GLP-1 produced by the strain	[41]
*Lactococcus lactis* MG1363 (pMG36e-GLP-1) with dopamine and β-glucan surface modification (L. lactis-GLP-1-DA@GN)	GLP-1(1–37). Dopamine and β-glucan were applied to the bacterial surface without altering the peptide sequence	Simulated gastrointestinal conditions and diabetic mice	Approximately 70 pg/mL GLP-1 in culture supernatant (ELISA) after 24–48 h	The surface coating improved bacterial survival in simulated gastrointestinal conditions. Oral administration improved measures of glucose and lipid metabolism in diabetic mice	The intact recombinant peptide was not identified in intestinal tissue	Serum GLP-1 immunoreactivity increased, but the assay could not distinguish recombinant peptide from host GLP-1	GLP-1R dependence was not tested	Coating influences bacterial survival, intestinal retention, and host responses. The separate contributions of GLP-1 expression, the bacterial strain and the surface coating were not determined	[28]
*Escherichia coli* Nissle 1917 (chromosomal GLP-1 cassette without an antibiotic selection marker)	GLP-1 encoded by a chromosomal cassette. The source sequence, peptide boundaries and processing were not specified	Mouse models of T1DM and T2DM	Recombinant expression was demonstrated without a quantitative culture concentration	After three weeks, fasting glucose in T2DM mice was 21.0 mmol/L after treatment versus 24.7 mmol/L in untreated diabetic controls. Glucose tolerance and pancreatic β-cell measures also improved. Glucose decreased in T1DM mice	Intact recombinant GLP-1 was not quantified in intestinal or other tissues	Portal and systemic recombinant GLP-1 measurements were not reported	The contribution of GLP-1R was not tested by receptor inhibition or genetic deletion	Parental EcN also reduced glucose in mice with T1DM. Metabolic outcomes alone cannot distinguish the contribution of recombinant GLP-1 from that of the bacterial strain	[42]
*Clostridium butyricum* (pMTL007-GLP-1)	GLP-1(1–37). The molecular form released in the intestine was not determined	Mice with T2DM	Approximately 84.58 pg/mL GLP-1 in culture supernatant	Lower blood glucose and lipid concentrations, with improved glucose tolerance and measures of insulin sensitivity	Intact recombinant GLP-1 was not measured after oral administration	Recombinant GLP-1 in portal or systemic blood was not quantified	Changes in GLP-1R expression and markers of AC and PKA activity were reported without pharmacological receptor inhibition	Butyrate production by the parental strain may contribute to the metabolic effects. Recombinant peptide exposure and plasmid stability were not quantified	[44]
*Escherichia coli* Nissle 1917 (GIFT-GLP-1, HexR circuit)	GLP-1(7–37). Expression was regulated by the HexR glucose sensor	GLP-1R reporter cells, diabetic mice and nonhuman primates	Glucose-dependent GLP-1 expression was measured in culture. Product concentrations were reported under conditions that differ from those used in other studies	Lower blood glucose and improved glycemic regulation in diabetic mice and nonhuman primates. Longer treatment was also associated with changes in circulating lipids and complications of diabetes	Circulating GLP-1 immunoreactivity was measured without specific identification of the bacterial product	Intact recombinant GLP-1 was not specifically identified in portal or systemic blood	GLP-1R activation was evaluated in reporter cells. Receptor inhibition or genetic deletion was not reported in treated animals	Glucose regulation of transgene expression was demonstrated, but systemic exposure to intact bacterial GLP-1 and receptor dependence of the metabolic effects were not measured	[43]
Modified GLP-1RAs and fusion proteins									
*Saccharomyces cerevisiae* SG2 (chromosomally integrated pNK-GLP construct)	Fusion protein containing 10 tandem repeats of a recombinant human GLP-1 analogue. Processing into active peptides was not examined	Hyperglycemic rats	Recombinant protein detected by immunoblotting. Approximately 0.84 mg/g packed cells after 24 h	Serum glucose decreased from 24.8 ± 1.40 to 21.2 ± 1.36 mmol/L following oral administration	Intact recombinant peptide was not measured in intestinal tissues	Portal and systemic recombinant peptide concentrations were not measured	GLP-1R dependence of the glycemic response was not examined	Viability of the lyophilized yeast preparation was not established. Intestinal release and systemic exposure to an intact receptor-active product were not examined	[45]
*Saccharomyces cerevisiae* LHY168 (chromosomally integrated pNK1-PGK-10laGLP-1 construct)	10 tandem repeats of a modified GLP-1 analogue containing A8S, K26Q and K34D substitutions intended to reduce proteolytic degradation	Mice with diabetes induced by an HFD and STZ	Intracellular protein detected by immunoblotting. Approximately 1.56 mg/g wet cells (ELISA)	Blood glucose decreased after 16 days of treatment. Reduced food and water consumption and attenuation of body weight loss were also reported	Intact recombinant peptide was not measured in intestinal contents or tissues	Portal and systemic recombinant peptide concentrations were not measured	GLP-1R dependence of the metabolic effects was not assessed	Intracellular protein expression did not establish intestinal release of an active peptide. Recombinant peptide exposure and receptor dependence were not examined	[47]
*Lacticaseibacillus paracasei* BL23 (pentameric constructs)	Pentamers containing GLP-1(7–37) derivatives with A8G substitution to reduce cleavage by DPP-4. One design also included K26Q and K34D to reduce degradation by trypsin. Linkers between monomers allowed trypsin processing	HIT-T15 cells, Goto–Kakizaki rats	Recombinant pentameric GLP-1 was detected by immunoblotting. Quantitative secretion yield was not reported	The processed product stimulated insulin secretion in HIT-T15 cells. Glucose tolerance did not differ significantly from that after parental bacteria, although nonfasting glucose decreased at selected time points	Intestinal concentrations of processed active peptide were not measured.	Portal and systemic exposure to recombinant peptide were not measured.	Insulin secretion was examined in cell culture without testing GLP-1R dependence in treated animals	The theoretical estimate of 0.3 µg GLP-1 per rat at 10^10^ CFU referred to monomeric surface display and did not represent a measured dose from the pentameric constructs. Intestinal peptide exposure was not quantified. Plasmid loss of approximately 40% and 65% was reported for the anchored and secreted variants, respectively	[32]
*Lacticaseibacillus paracasei* NFBC 338 (pNZ44-KGLP-1)	KGLP-1, an analogue of GLP-1(7–37) designed to resist degradation by DPP-4 while retaining insulinotropic activity	Rats and mice fed an HFD in separate 21-day and 12-week experiments, respectively	Approximately 1.5 ng/mL KGLP-1 in culture medium, measured by ELISA	In rats, triglycerides and LDL cholesterol decreased relative to the isogenic control strain, without significant improvement in glucose tolerance. In mice, glucose metabolism improved relative to untreated HFD controls, but glycemic outcomes did not differ significantly from those after the isogenic bacterial strain	Intact KGLP-1 was not measured in intestinal tissue or blood after oral administration	Portal and systemic KGLP-1 concentrations were not measured	Biological activity was evaluated in cell culture. GLP-1R dependence was not tested in treated animals	Several changes in adiposity and metabolism also occurred with the isogenic bacterial control, preventing attribution of these effects solely to KGLP-1	[34]
*Escherichia coli* Nissle 1917 (pGEX-GLP-1 GM)	GLP-1 GM, a modified GLP-1(7–37) sequence	Mice fed an HFD	Recombinant expression was reported without a quantitative measurement in culture medium	Lower food intake, weight gain and adiposity, with improved glucose tolerance and changes in hepatic lipid metabolism	The recombinant peptide was not measured in target tissues	Portal and systemic concentrations of GLP-1 GM were not measured	Hypothalamic neuropeptide transcripts were measured, but GLP-1R dependence was not tested	Hypothalamic transcript changes and reduced food intake do not identify the anatomical site of recombinant peptide action	[40]
*Lactococcus lactis* (A8SGLP-1 expression construct)	Analogue of GLP-1(7–36) with A8S substitution intended to reduce degradation by DPP-4. *C*-terminal processing was not characterized	In vitro testing of DPP-4 resistance and db/db mice	Expression was reported without a quantitative measurement of recombinant peptide output	Improved glycemic measures and reduced fat mass. Total body weight did not decrease	The A8S peptide was not distinguished analytically from endogenous GLP-1	Circulating GLP-1 immunoreactivity was measured without identifying the recombinant A8S peptide	Resistance to DPP-4 was examined in vitro. GLP-1R dependence was not tested in treated mice	The GLP-1 immunoassay did not discriminate recombinant A8SGLP-1 from endogenous hormone	[26]
*Saccharomyces boulardii* (integrated exendin-4 construct)	Exendin-4 (39 amino acids)	Isolated pancreatic islets and HFD-fed mice exposed to cold	Approximately 15 nM per OD_600_ in aerobic culture and 11 nM per OD_600_ in anaerobic culture	During cold exposure (8 °C), food intake decreased by approximately 25%, and body weight loss was approximately fourfold greater than in mice receiving the control yeast strain	Exendin-4 immunoreactivity was detected in portal plasma, but the intact molecular form was not identified	Portal peptide immunoreactivity was observed in a subset of animals. The assay did not establish the presence of intact exendin-4	Experiments with pancreatic islets from mice lacking GLP-1R supported receptor dependence in vitro. Receptor inhibition was not evaluated in treated animals	Metabolic effects were observed during cold exposure but not at room temperature. GLP-1R dependence of the response was not examined in treated mice	[48]
*Lactiplantibacillus plantarum* WCFS1 (pSIP403-p11-0373-mGLP-1)	Analogue of human GLP-1(7–36) containing A8G to resist cleavage by DPP-4 and K26Q and K34D to reduce cleavage by trypsin. The protein detected in culture medium retained the secretion signal peptide	Analysis of recombinant peptide in culture and db/db mice	An approximately 9 kDa product retaining the secretion signal was detected by immunoblotting. Smaller products appeared after trypsin treatment. Quantitative yield was not reported	Improved glucose tolerance and indices of pancreatic β-cell function in db/db mice	Processed active mGLP-1 was not identified in intestinal contents after oral administration	Intact mGLP-1 was not measured in portal or systemic blood	Biological activity was examined in vitro. GLP-1R dependence was not tested in treated mice	Processing of the fusion product in the intestine was not measured. The plasmid contained an erythromycin resistance marker	[38]
*Lactococcus lactis* NZ9000 (expression regulated by blue light)	GLP-1(7–37), expressed under optical regulation. A separate construct encoded a GLP-1 fusion with an Fc domain intended to prolong peptide persistence	Optical induction in culture and diabetic rodent models	Approximately 4000 pg/mL GLP-1 after 15 min of illumination (ELISA).	Optical induction of microbial GLP-1 expression was accompanied by improvements in glucose and body weight outcomes in treated rodents	The intact bacterial peptide was not measured in intestinal tissue	Portal and systemic exposure to recombinant peptide was not quantified	Regulation of expression by light was demonstrated. GLP-1R dependence was not examined in treated rodents	The system required external light and additional optical components. Peptide processing and systemic exposure were not measured	[27]
*Lactobacillus gasseri Lgs* ^GPA^	Fusion of a GLP-1 sequence with a PTD intended to facilitate epithelial uptake and an ABP to prolong exposure	MIN6 cells, db/db mice and separate experiments using purified fusion protein	The fusion product was detected in culture supernatant by immunoblotting and GLP-1 ELISA	Culture supernatant increased insulin secretion and PDX-1 expression in MIN6 cells. Oral administration reduced hyperglycemia, measures of insulin resistance and dyslipidemia in db/db mice	GPA was detected in rat blood by HPLC and immunoblotting following oral administration of the engineered bacteria	Qualitative detection of GPA in blood was reported after oral bacterial administration. Systemic peptide concentrations and pharmacokinetic parameters were not determined	Insulin secretion was examined in MIN6 cells. GLP-1R dependence was not tested in treated mice	Epithelial uptake and binding to albumin were not demonstrated following oral bacterial administration. Injection experiments using purified peptide do not establish exposure after bacterial treatment	[31]
*Lactococcus lactis* NZ3900 (pLN-LGLP or pLN-EX4, inducible by nisin)	LGLP, an analogue of GLP-1(7–36) with A8G. The alternative product was exendin-4 (39 amino acids)	INS-1 cells, polarized epithelial models, Ussing chambers, intestinal loop experiments and db/db mice	Approximately 180 pg/mL LGLP and 130 pg/mL exendin-4 after 24 h of nisin induction (ELISA).	Oral administration of LL-LGLP improved glycemic measures and pancreatic islet morphology. Experiments with purified fluorescent peptides showed intestinal uptake	An LGLP-specific peptide fragment was detected in peripheral plasma by LC–MS following oral administration of the engineered bacteria	LC–MS confirmed the presence of a fragment specific to recombinant LGLP in peripheral plasma. Intact active LGLP was not quantified	GLP-1R competition reduced uptake of purified fluorescent peptides by epithelial cells. GLP-1R dependence of the metabolic response was not evaluated in mice	Detection of an LGLP fragment supports intestinal absorption of bacterial product but does not establish systemic exposure to intact active LGLP	[29]
Stimulation of endogenous GLP-1 secretion									
*Lactococcus lactis* P11P9 (P9 secretion construct)	P9 (Amuc_1631), a protein originating from *Akkermansia muciniphila* that stimulates secretion of host GLP-1. Interaction with ICAM-2 has been proposed as part of the mechanism	Human colorectal adenocarcinoma NCI-H716 cells, and HFD-fed mice	P9 secretion was detected without a quantitative concentration of recombinant protein	GLP-1 secretion increased 2.65 times in NCI-H716 cells. Food intake and weight gain decreased in treated mice	Host GLP-1 was measured. The tissue distribution of recombinant P9 was not investigated	Serum GLP-1 concentrations increased after administration of the engineered strain. Circulating P9 was not measured	Increased intestinal expression of genes involved in GLP-1 synthesis was reported. The contribution of ICAM-2 and GLP-1R to the metabolic effects was not tested by receptor inhibition in treated mice	The relevant intestinal pool of ICAM-2 and its contribution to the effects of bacterial P9 were not examined directly	[58]

Abbreviations: ABP, albumin-binding peptide; AC, adenylyl cyclase; CFU, colony-forming unit; DPP-4, dipeptidyl peptidase-4; EcN, *Escherichia coli* Nissle 1917; GLP-1, glucagon-like peptide-1; GLP-1R, glucagon-like peptide-1 receptor; HFD, high-fat diet; ICAM-2, intercellular adhesion molecule 2; MDCK, Madin–Darby canine kidney; PKA, protein kinase A; PTD, protein transduction domain; STZ, streptozotocin; T1DM, type 1 diabetes mellitus; T2DM, type 2 diabetes mellitus.

**Table 2:** Translational requirements and validation priorities for GLP-1-oriented living therapeutics.

Translational Issue	Development or Evaluation Approach	Experimental Validation	Experimental Status	References
Limited intestinal survival and local activity	Chassis selection, protective formulation, surface modification, and repeated administration	Microbial viability, intestinal distribution, residence time, and local recombinant product formation	Surface modification improved bacterial survival under simulated gastrointestinal conditions. Bacterial distribution was investigated using modified EcN. Regional production of functional recombinant peptides remains insufficiently characterized	[28,41,100,101]
Limited exposure of the recombinant product at the intended target	Peptide stabilization, appropriate secretion or release systems, and characterization of intestinal transport	Molecular identity, proteolytic processing, tissue localization, epithelial transport, and receptor-dependent activity	A construct-specific peptide fragment was identified in peripheral blood. Qualitative detection of a recombinant fusion protein was reported, whereas portal exendin-4 immunoreactivity was observed in another study. Quantitative systemic exposure to intact, biologically active recombinant agonists remains insufficiently characterized	[29,31,48]
Uncertain mechanism of endogenous GLP-1 stimulation	Characterization of recombinant microbial effectors and their interactions with enteroendocrine cells	Host GLP-1 secretion, involvement of the proposed upstream target, and dependence of downstream effects on GLP-1R signaling	Recombinant P9 stimulated GLP-1 secretion in cellular and metabolic animal models, although involvement of the proposed upstream target was not directly established. HldSE-mediated effects were attenuated by GLP-1R blockade in a non-metabolic colitis model	[58,114]
Insufficient control of recombinant expression	Light-responsive or metabolically regulated expression systems	Basal expression, induction threshold, dynamic range, response kinetics, reversibility, and recombinant product output	Light-regulated and glucose-responsive expression systems were evaluated in GLP-1-oriented platforms. Quantitative relationships between regulatory inputs, active peptide exposure, and pharmacodynamic responses remain incompletely characterized	[27,43]
Genetic and functional instability	Chromosomal integration, optimized integration sites, and reduced expression burden	Genetic integrity, plasmid retention, transcript and protein abundance, functional activity, and microbial fitness during serial passage	Plasmid loss was reported in GLP-1-expressing strains. An integrated GLP-1 sequence remained intact after serial passage despite reduced transcript abundance. Long-term functional stability following intestinal administration remains insufficiently characterized	[32,35,122,123]
Antibiotic-resistance markers and transferable genetic elements	Marker-free genetic modification and reduced dependence on mobile elements or antibiotic selection	Genetic characterization, resistance determinants, mobile elements, and horizontal gene transfer	Chromosomal integration without an antibiotic selection marker was reported in a GLP-1-producing EcN strain, whereas another expression system contained an erythromycin-resistance marker. Horizontal transfer of recombinant genetic material has not been systematically evaluated in GLP-1-oriented platforms	[38,42,53,134]
Unpredictable microbial persistence and limited reversibility	Transiently persistent strains, auxotrophy, conditional elimination, and regulated expression	Microbial clearance, fecal shedding, cessation of recombinant product formation, persistence of pharmacodynamic activity, and containment escape	Intestinal persistence was investigated in a glucose-responsive GLP-1 platform. Clinical microbial clearance was evaluated for SYNB1618, while auxotrophy and conditional elimination were investigated in living therapeutics for other indications. Corresponding containment and functional washout data for GLP-1-oriented platforms remain limited.	[43,126,127,128,132,133]
Chassis- and formulation-dependent biological effects	Parental, isogenic, product-negative, and formulation-matched controls	Comparative biological responses under matched microbial doses and experimental conditions	Parental or isogenic strains produced metabolic effects in individual studies. Surface modification introduced additional experimental variables, while endogenous butyrate production may contribute to the effects of engineered *C. butyricum*	[28,34,42,44]
Uncertain relationship between microbial dose and pharmacological activity	Quantitative product characterization and mechanism-specific potency assays	Administered CFU, functional product formation, biological potency, and pharmacodynamic responses	Recombinant peptide concentrations and microbial doses were reported using heterogeneous methods. Estimates of peptide output per CFU have not been consistently linked to intestinal exposure or pharmacodynamic activity following microbial administration	[23,29,32,43]
Immunogenicity of recombinant products and microbial components	Characterization of non-native peptide sequences, fusion domains, and host immune responses	Anti-product and anti-chassis antibodies, neutralizing activity, hypersensitivity, and repeated-dose immune responses	Modified GLP-1 sequences and fusion proteins have been investigated, but their immunogenicity following repeated administration of the corresponding engineered microorganisms has not been systematically characterized	[27,31,39,48]
Interindividual variation in intestinal physiology and microbiota	Assessment of host-dependent microbial persistence and functional activity	Microbial colonization, recombinant product formation, and pharmacodynamic responses across different host and microbiota backgrounds	Host-dependent intestinal colonization has been demonstrated for unmodified probiotics. Predictive relationships between host characteristics and therapeutic responses have not been established for GLP-1-oriented living therapeutics	[141]
Manufacturing and storage variability	Defined cell banking, fermentation, formulation, storage conditions, and product-specific quality specifications	Strain identity, purity, viable cell concentration, genetic stability, biological potency, and storage stability	Manufacturing and quality-control requirements have been described for live biotherapeutic products generally. Clinically validated potency specifications for GLP-1-oriented engineered microorganisms have not been established in the studies reviewed	[53,135,136]

Note: The experimental status distinguishes findings obtained in GLP-1-oriented engineered microorganisms from those reported for living therapeutics developed for other indications. Studies outside the GLP-1 field are included only where they provide relevant experimental evidence concerning microbial engineering, containment, or translational evaluation. CFU, colony-forming units; EcN, *Escherichia coli* Nissle 1917; GLP-1, glucagon-like peptide-1; GLP-1R, GLP-1 receptor.

## Data Availability

Data sharing is not applicable. No new data were created or analyzed in this study.

## Abbreviations

The following abbreviations are used in this manuscript:

ABP	albumin-binding peptide
AC	adenylyl cyclase
AKT	protein kinase B
AUC	area under the curve
cAMP	cyclic adenosine monophosphate
CFU	colony-forming unit
CRISPR	clustered regularly interspaced short palindromic repeats
DPP-4	dipeptidyl peptidase-4
EcN	*Escherichia coli* Nissle 1917
ELISA	enzyme-linked immunosorbent assay
ENS	enteric nervous system
FDA	U.S. Food and Drug Administration
FFAR2	free fatty acid receptor 2
FFAR3	free fatty acid receptor 3
FXR	farnesoid X receptor
GCG	glucagon
GLP-1	glucagon-like peptide-1
GLP-1R	glucagon-like peptide-1 receptor
GLP-1RA	glucagon-like peptide-1 receptor agonist
GPBAR1	G protein-coupled bile acid receptor 1
HFD	high-fat diet
HPLC	high-performance liquid chromatography
ICAM-2	intercellular adhesion molecule 2
IL	interleukin
LC–MS	liquid chromatography–mass spectrometry
LPS	lipopolysaccharide
PAMPs	pathogen-associated molecular patterns
PKA	protein kinase A
PI3K	phosphoinositide 3-kinase
PTD	protein transduction domain
PYY	peptide YY
RT-qPCR	reverse transcription quantitative polymerase chain reaction
SCFA	short-chain fatty acid
siRNA	small interfering RNA
SNAC	salcaprozate sodium
STZ	Streptozotocin
T1DM	type 1 diabetes mellitus
T2DM	type 2 diabetes mellitus
TGR5	Takeda G protein-coupled receptor 5
